# AhfA is required for mycolic acid homeostasis and cell envelope integrity in Corynebacteriales

**DOI:** 10.1099/acmi.0.001208.v3

**Published:** 2026-10-12

**Authors:** Mamata Modak, Karin Krumbach, Jan Marienhagen, Apoorva Bhatt, Gurdyal S. Besra

**Affiliations:** 1 School of Biosciences and Institute of Microbiology and Infection, University of Birmingham, Edgbaston, Birmingham B15 2TT, UK; 2 Institute of Bio- and Geosciences, IBG-1: Biotechnology, Forschungszentrum Jülich, D-52425 Jülich, Germany; 3 Institute of Biotechnology, RWTH Aachen University, Worringer Weg 3, D-52074 Aachen, Germany

**Keywords:** cell envelope, *Corynebacterium*, mycolic acid biosynthesis

## Abstract

Mycolic acids are critical components of the *Corynebacteriales* cell envelope, contributing to cellular morphology, permeability and antibiotic resistance. While the core enzymes responsible for mycolic acid synthesis are relatively well described, little is known about the accessory factors that coordinate their biosynthesis and integration into the cell envelope. In this study, we investigated the role of AhfA in *Corynebacterium glutamicum* using genetic, biochemical and physiological approaches. Loss of AhfA resulted in broad effects on cell growth, morphology, lipid composition and cell envelope integrity, which were restored upon complementation. Our analysis suggests that AhfA acts as an early stage of mycolic acid synthesis supporting the formation of the lipid-rich outer cell envelope. Notably, a similar reduction in mycolic acid-containing glycolipids was observed in mycobacterial species, suggesting that the role of AhfA in mycolic acid metabolism is conserved across *Corynebacteriales*. This conservation suggests that accessory proteins involved in mycolic acid biosynthesis and homeostasis may represent potential targets for therapeutic intervention. These findings identify AhfA as a previously uncharacterized factor in cell envelope assembly and highlight the complexity of lipid integration in *Corynebacteriales*.

## Data Summary

All data supporting the findings of this study are contained within the article and its supplementary materials.

## Introduction

Members of the *Corynebacteriales* including *Corynebacterium*, *Mycobacterium*, *Nocardia* and *Rhodococcus* have a uniquely structured cell envelope. These bacteria produce a complex, multilayered cell envelope that plays a key role in their natural resistance to many antibiotics and their ability to cause disease [1]. The mycobacterial and corynebacterial cell envelopes consist of an inner membrane (IM), a layer made up of covalently linked peptidoglycan, arabinogalactan and long-chain mycolic acids and an outer membrane (OM) composed of mycolic acids attached to arabinogalactan [2]. The defining characteristic of the *Corynebacteriales* is the presence of mycolic acids. Mycolic acids are long-chain α-alkyl, β-hydroxy fatty acids, 70–90 carbons in length in pathogenic species, such as *Mycobacterium tuberculosis*, and shorter 30–36 carbons in the industrially important species *Corynebacterium glutamicum* [3]. They play a pivotal role in shaping cell surface properties, such as hydrophobicity and mechanical strength, which are essential for maintaining morphology and overall cell envelope integrity [4]. Disruption of mycolic acid synthesis through enzymatic blockade or genetic alterations results in slowed growth, increased vulnerability to stresses and morphological defects, such as elongated cells or cell lysis [5, 6].

Mycolic acid biosynthesis and transfer in mycobacteria and corynebacteria involve conserved yet specialized enzymatic machinery that assembles these long-chain fatty acids into the cell wall arabinogalactan to form the protective mycomembrane [5]. In mycobacteria, fatty acid synthase I (FAS-I) initiates meromycolate chain elongation (C20 to C60), which is further processed by the fatty acid synthase II dissociable enzymes: β-ketoacyl synthases KasA/B, reductases MabA and InhA, dehydratase and the pivotal condensing polyketide synthase Pks13 that catalyses the Claisen-type condensation of α- and meromycolyl chains, followed by maturation via β-hydroxyacyl reductases and cyclopropane synthases for α-, methoxy- and keto-mycolates with transfer mediated by the antigen 85 complex of mycolyltransferases that esterify mycolates to trehalose or arabinogalactan [5, 7–10]. Corynebacteria synthesize shorter corynomycolates (~ C36) via an analogous FAS-I priming mechanism but rely on distinct acyl carboxylases, comprising shared biotinylated α-subunits AccBC and AccE paired with β-subunits AccD2/AccD3 [11–13], alongside Pks13 for final condensation [13, 14] and transferases that link corynomycolates to trehalose monomycolates or cell wall-linked arabinogalactan [11, 13].

Despite significant advances in elucidating the core enzymatic machinery for mycolic acid biosynthesis in *Corynebacteriales*, our understanding of the regulatory and accessory proteins that govern mycolic acid biosynthesis, processing and integration into the cell envelope remains limited. Although regulators like FasR have been shown to control upstream *fas* and *acc* gene expression [15], not much is known about regulatory factors and accessory factors involved in mycolic acid biosynthesis or downstream stages of mycolate maturation, transport and covalent attachment to trehalose monomycolates or the arabinogalactan–peptidoglycan complex. Genomic analyses identified conserved candidates for lipid-binding chaperones, flippases and scaffold proteins in corynebacteria. These include homologues of mycobacterial TtfA and MSMEG_5308. These genes are thought to aid mycolate flipping, mycomembrane organization and coordination with cell division or stress responses [16]. In mycobacteria, TtfA acts as an essential accessory protein that complexes with the MmpL3 transporter for trehalose monomycolate export across the plasma membrane [16, 17], whereas MSMEG_5308 is non-essential but accumulates during envelope stress to stabilize the MmpL3/TtfA transport machinery [16]. However, only a few have been functionally validated. Complementing these studies, recent functional screens have begun to uncover distinct factors critical for envelope integrity [18]. Addressing these gaps is essential to clarify how cell envelope biogenesis is temporally and spatially regulated during growth, division and adaptation with broader implications for microbial fitness, antibiotic resistance and pathogenesis in actinobacteria.

One such poorly characterized factor is AhfA. Previous high-throughput transposon mutagenesis screens identified AhfA as a critical component required for mycolic acid biosynthesis and cell envelope integrity [18]. However, these studies relied on insertional mutants in which polar effects and heterogeneous disruption alleles complicated functional interpretation and left the precise molecular role of AhfA unresolved. To overcome these limitations and to gain a clearer understanding of its function, in this study, a defined chromosomal knockout mutant was generated, allowing an unambiguous link to specific phenotypes. The mutant also provided a platform for genetic complementation and structure–function analysis. In *C. glutamicum*, the *ahfA* locus is embedded within a genomic region (Fig. 1) that also harbours genes encoding a fatty acid CoA ligase and a UDP-*N*-acetylmuramate dehydrogenase alongside several conserved but uncharacterized ORFs. The composition of this locus suggests that AhfA may participate in processes associated with cell envelope metabolism. The close genomic proximity of *ahfA* to genes involved in fatty acid activation and peptidoglycan precursor modification further supports the hypothesis that AhfA may operate at the interface of lipid and carbohydrate biogenesis within the cell wall, providing a strong rationale to investigate its precise biochemical role in mycolic acid assembly.

**Fig. 1.**
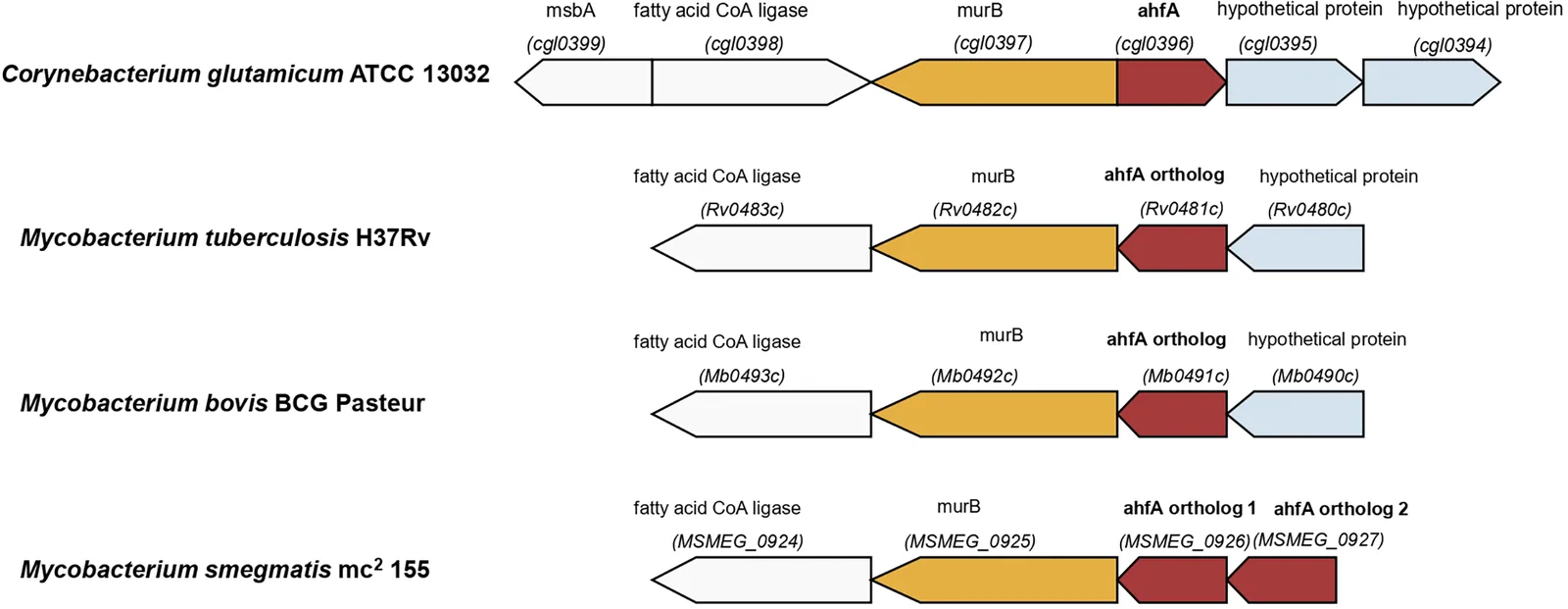
Genomic organization of *ahfA* and neighbouring genes in *Corynebacterium* and *Mycobacterium* species. Schematic comparison of the genomic loci surrounding *ahfA* in *C. glutamicum* ATCC 13032, *M. tuberculosis* H37Rv, *Mycobacterium bovis* BCG Pasteur and *Mycobacterium smegmatis* mc²155. Genes are represented by arrows indicating the direction of transcription. *ahfA* and its orthologues are highlighted in red. Genes are coloured according to homology.

**Fig. 2.**
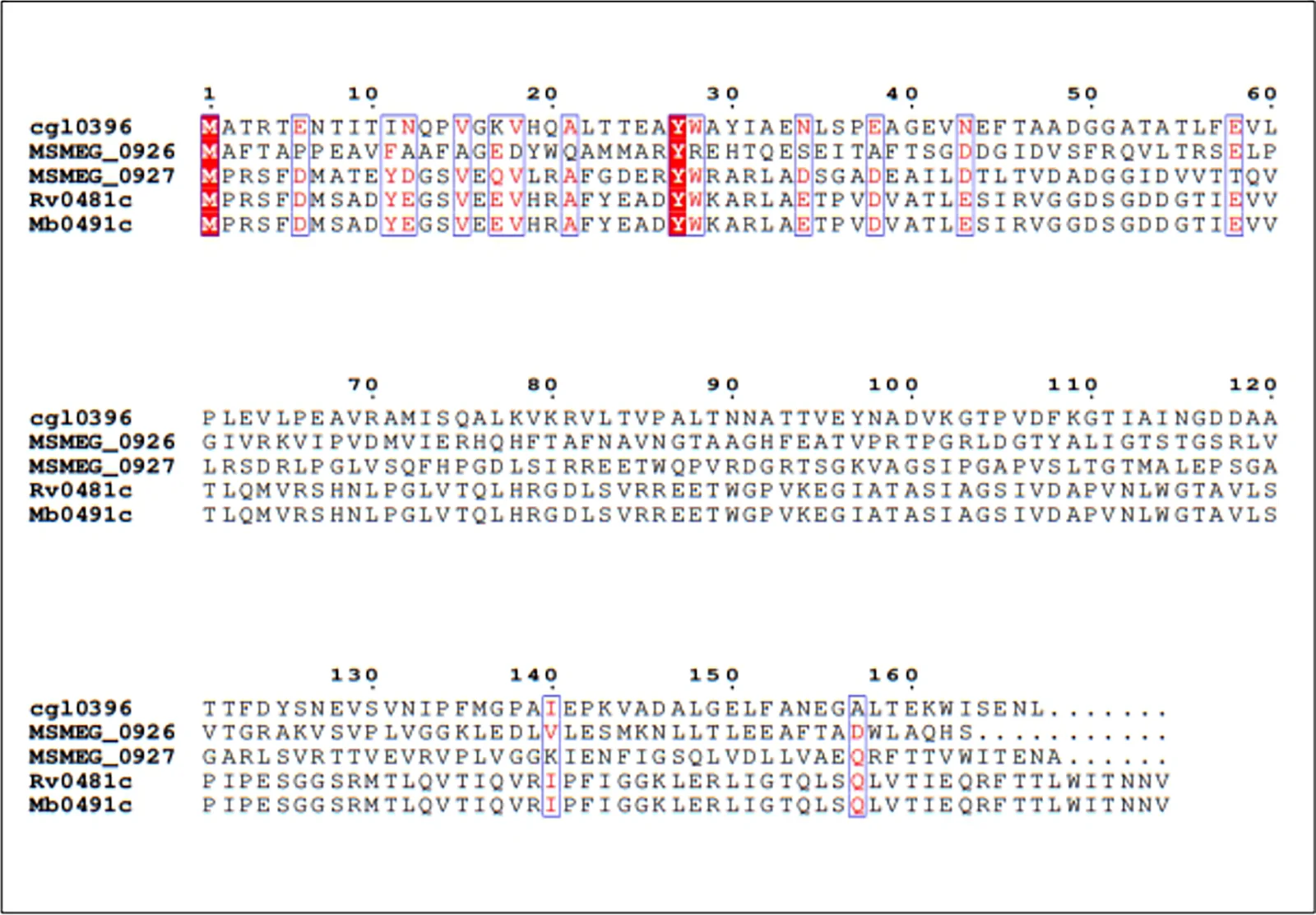
Alignment of *ahfA* amino acid sequences from *C. glutamicum* along with orthologues from *M. smegmatis*, *M. tuberculosis* and *M. bovis* BCG. Sequences were aligned using ClustalW and rendered with ESPript.

Notably, AhfA is conserved across *Corynebacterium* and *Mycobacterium* species [18]. This indicates an evolutionarily conserved role in actinobacterial envelope biology (Figs. 1 and 2).

In this study, we investigated the functional role of AhfA through an integrated genetic, biochemical and physiological analysis in *C. glutamicum* and its mycobacterial orthologues. Using defined genomic mutants combined with detailed phenotypic characterization, we sought to elucidate the contribution of AhfA to cell envelope biogenesis, integrity and homeostatic regulation within the order *Corynebacteriales*.

## Methods

### Bacterial strains and culture conditions

The following strains were used in this study, *C. glutamicum* ATCC 13032 was cultivated in Brain Heart Infusion Soribtol (BHIS) medium composed of 37 g l^−1^ brain heart infusion and 91 g l^−1^ sorbitol. Cultures were incubated at 30 °C under shaking conditions. *Mycobacterium smegmatis* mc^2^155 strains were grown in tryptic soy broth supplemented with 0.05% (v/v) Tween 80 and incubated at 37 °C with shaking. *Mycobacterium bovis* BCG (Pasteur) cultures were maintained in Middlebrook 7H9 medium supplemented with 0.2% (v/v) glycerol and 0.05% (v/v) Tween 80 and incubated at 37 °C under static conditions.

### In-frame deletion of *ahfA* in *C. glutamicum* ATCC 13032 and construction of pEKEx2_cgl0396 for complementation experiments

All oligonucleotides for in-frame deletion of *ahfA* (cgl0396) or episomal expression of *ahfA* were synthesized by Eurofins Genomics (Ebersberg, Germany) and are listed in Table 1. For molecular cloning work, standard protocols, e.g. PCR and Gibson Assembly, were used [19, 20]. All strains used are listed in Table 2. Plasmid DNA was isolated using the NucleoSpin Plasmid kit (Macherey-Nagel, Dueren, Germany), and verification of the constructed plasmids was performed either by restriction analysis using restriction enzymes from Thermo Scientific (Schwerte, Germany) or by colony PCR using the DreamTaq Green PCR Master Mix (2x) containing DreamTaq DNA Polymerase from Thermo Scientific (Schwerte, Germany). DNA sequencing was conducted at Eurofins Genomics (Ebersberg, Germany). *E. coli* DH5α, routinely used for cloning purposes, was transformed using the RbCl method, whereas *C. glutamicum* was always transformed by electroporation followed by an additional heat shock at 46 °C for 6 min [21, 22]. In-frame deletion was performed by two-step homologous recombination using the plasmid pk19*mobsacB-*Δcgl0936 as previously described [23], and successful deletion of *ahfA* was confirmed (Fig. S1, available in the online Supplementary Material).

**Table 1.** Oligonucleotides used in this study for *C. glutamicum* constructs

Name	DNA sequence (5′–3′)	
**Construction of pk19*mobsacB-*Δcgl0396**		
pdcgl0396_Nout	GCAGGTCGACTCTAGAGGCGGGTGAGTACATCGGAAACTT	
pdcgl0396_Nin	GGGTAGGTGATTTGAATTTGTTTCTGTACGCGTTGCCATG	
pdclg0396_Cin	ACAAATTCAAATCACCTACCCGCTCTGACCGAAAAGTGG	
pdcgl0396_Cout	CGAGCTCGGTACCCGGGCTTCGAAAAACAGTCCGCCT	
**Colony-PCR/sequencing of pk19*mobsacB-*Δcgl0396**		
pdcgl0396_proof-fwd	ACCCAGGAGACTTGGTGGGT	
pdclg0396_proof-rev	GGTGCAGCGTTTGCCGATTTCATC	
**Construction of pEKEx2_cgl0396**		
pEx-cgl0396_fwd2	CAGGTCGACTCTAGAGAAGGAGATATACATATGGCAACGCGTACAGAAAACACCA	
pEx-cgl0396_rev	TCGAGCTCGGTACCCGGGTTATTAGAGGTTCTCGGAGATC	
**Colony-PCR/sequencing of pEKEx2_cgl0396**		
pEKEx-for2	cggcgtttcacttctgagttcggc	
pEKEx-rv2	gatatgaccatgattacgccaagc	

**Table 2.** Strains and plasmids used in this study for *C. glutamicum*

Strain or plasmid	Relevant characteristic	Source or reference
***E. coli* strain**		
DH5α	F– Φ80*lacZ*ΔM15 Δ(*lacZYA-argF*)U169 *recA1 endA1 hsdR17* (rK–, mK+) *phoA supE44* λ– *thi-1 gyrA96 relA1*	Invitrogen (Karlsruhe, Germany)
***C. glutamicum* strains**		
ATCC 13032 (WT)	Biotin auxotroph wild-type strain	[34]
Δ*ahfA*	WT with in-frame deletion of cgl0396	This work
**Plasmids**		
pk19*mobsacB-*Δcgl0396	*kan*^r^; plasmid for in-frame deletion of cgl0396	This work
pEKEx2	*kan*^r^; *E. coli–C. glutamicum* shuttle vector for regulated gene expression (*lacI^q^*, P*tac*, *lacO*; pBL1ori*Cg*; ColE1ori*Ec*)	[35]
pEKEx2_cgl0396	*kan*^r^; pEKEx2 derivative for the regulated expression of cgl0396 from *C. glutamicum* ATCC 13032	This work

*kan*r: kanamycin resistance.

For complementation experiments, plasmid pEKEx2_cgl0396 for IPTG-inducible episomal expression of *ahfA* was constructed and transformed into *C. glutamicum* Δ*ahfA*. Chromosomal DNA from *C. glutamicum* ATCC 13032 was used as a template to amplify the ORF of *ahfA*.

### Generation of *M. smegmatis* and *M. bovis* BCG knockouts using specialized transduction

Allelic exchange substrates (AESs) and specialized transducing phages were generated as previously described in Jain *et al*. [24]. Around 1 kb regions upstream and downstream of the target gene were PCR amplified using gene-specific primers incorporating restriction sites and digested with the appropriate enzyme. All oligonucleotides used are listed in Table 3.

**Table 3.** Oligonucleotides used in this study for *Mycobacterium* constructs

Name	DNA sequence (5′–3′)	Enzyme used
**Construction of Δ*MSMEG0926***		
LL	TTTTTTTTGCATAAATTGCTGGTGTCGCAGTTCCATC	BstAPI
LR	TTTTTTTTGCATTTCTTGC GTTGAACGCGGTGAAGTG	BstAPI
RL	TTTTTTTTCCATAGATTG TATGCGCTCATCGGGACCTC	Van91I
RR	TTTTTTTTCCATCTTTTGG GGACTCGACCAGCTCCAGATTC	Van91I
**Construction of Δ*MSMEG0927***		
LL	TTTTTTTTCCATAAATTGG AGCGCATGCTTGGTGGACAG	Van91I
LR	TTTTTTTTCCATTTCTTGG TGCGTGGTCACCACGTCGATAC	Van91I
RL	TTTTTTTTGCATAGATTGC TCGGCGGCAAGATCGAGAAC	BstAPI
RR	TTTTTTTTGCATCTTTTGC CCGTATCCGAGGTCGATCAC	BstAPI
**Construction Δ*Mb0491c***		
LL	TTTTTTTTCCATAAATTGG AGAACGATCCCACGCTCCAG	Van91I
LR	TTTTTTTTCCATTTCTTGG ATCGATCCCGCAATGGA	Van91I
RL	TTTTTTTTGCATAGATTGC GTCACCATCCAGGTGCGTATCC	BstAPI
RR	TTTTTTTTGCATCTTTTGC GTTGGTCACCCACGCGATTG	BstAPI

Construction of Δ*MSMEG0926+MSMEG0927.*

Use LL/LR of *MSMEG0927* and RL/RR of *MSMEG0926.*

Digested flanking fragments were ligated with Van91I-treated p0004S, which carries the hygromycin resistance and *sacB* counter selection cassette. Recombinant plasmids were transformed into *Escherichia coli* TOP10 selected on hygromycin and verified by digestion followed by sequencing analysis. For phasmid construction, sequence-confirmed AES plasmids were linearized with PacI and ligated into PacI-digested temperature-sensitive mycobacteriophage phAE159. Ligation products were packaged and used to infect *E. coli* HB101 to recover recombinant phasmids. These were confirmed by PacI digestion. High-titre phage lysates were prepared by infecting *M. smegmatis* and *M. bovis* BCG, overlaying infected cultures in top agar and harvesting plaques after incubation at 30 °C. Phage lysates were filtered and titrated to obtain stocks ≥10¹⁰ p.f.u. ml^−1^. Specialized transduction was performed by incubating washed mid-log phase mycobacterial cells with high-titre recombinant phage at the non-permissive temperature (37 °C) to allow allelic exchange without further phage replication. Following recovery in supplemented medium, cells were plated on hygromycin-containing agar to select recombinant clones. Hygromycin-resistant colonies were screened to confirm gene replacement using whole-genome sequencing and PCR (Fig. S2), yielding the desired deletion mutants. The following mutants were generated: Δ*MSMEG0926*, Δ*MSMEG092*7, Δ*MSMEG0926*+Δ*MSMEG0927* (double mutant) and Δ*Mb0491c*. All strains used are listed in Table 4.

**Table 4.** Strains and plasmids used in this study for *Mycobacterium*

Strain or plasmid	Relevant characteristic	Source or reference
**Plasmids**		
p0004s	Cloning vector for engineering allelic exchange substrates for specialized transduction	Lab stock
pΔ*MSMEG0926*	Derivative of p0004s designed for allelic exchange of *MSMEG0926*	This work
pΔ*MSMEG0927*	Derivative of p0004s designed for allelic exchange of *MSMEG0927*	This work
pΔ*MSMEG0926+MSMEG0927*	Derivative of p0004s designed for allelic exchange of *MSMEG0926+MSMEG0927*	This work
pΔ*Mb0491c*	Derivative of p0004s designed for allelic exchange of *Mb0491c*	This work
***M. smegmatis* strains**		
mc^2^155	Electroporation-proficient *ept* mutant of *M. smegmatis* strain mc^2^6	[36]
Δ*MSMEG0926*	Deletion mutant of mc^2^155 in which *MSMEG0926* is replaced by *hyg*	This work
Δ*MSMEG0927*	Deletion mutant of mc^2^155 in which *MSMEG0927* is replaced by *hyg*	This work
Δ*MSMEG0926+*Δ*MSMEG0927*	Deletion mutant of mc^2^155 in which *MSMEG0926* and *MSMEG0927* are replaced by *hyg*	This work
***M. bovis* BCG strains**		
*M. bovis* BCG	Pasteur substrain of *M. bovis* BCG	[37]
Δ*Mb0491c*	Deletion mutant of *M. bovis* in which *Mb0491c* is replaced by *hyg*	This work
**Phages**		
phAE159	Conditionally replicating shuttle phasmid derived from the lytic mycobacteriophage TM4	[37]
phΔ*MSMEG0926*	Derivative of phAE159 obtained by cloning pΔ*MSMEG0926* into its unique PacI site	This work
phΔ*MSMEG0927*	Derivative of phAE159 obtained by cloning pΔ*MSMEG0927* into its unique PacI site	This work
phΔ*MSMEG0926+MSMEG0927*	Derivative of phAE159 obtained by cloning pΔ*MSMEG0926+MSMEG0927* into its unique PacI site	This work
phΔ*Mb0491c*	Derivative of phAE159 obtained by cloning pΔ*Mb0491c* into its unique PacI site	This work
***E. coli* strains**		
HB101	General cloning host; used for propagating allelic-exchange plasmids	Lab stock
TOP10	General cloning host; used for routine plasmid propagation	Lab stock

### Phase-contrast microscopy of *C. glutamicum* strains

Live cells of *C. glutamicum* wild-type, Δ*ahfA* and Δ*ahfA* pEKEx2_cgl0396 strain were prepared for microscopy by spotting 5 µl of an actively growing culture onto a clean microscope slide followed by placement of a coverslip. The edges of the coverslip were sealed with nail varnish to prevent evaporation and maintain cell viability during imaging. Samples were examined by phase-contrast microscopy using a 100× oil immersion objective on a Nikon E600 compound microscope. Images were acquired using either a Zeiss LSM 510 confocal microscope with the associated Zeiss software or a Nikon DXM1200 digital camera operated through Nikon ACT software [25]. Fiji ImageJ software was used to measure the length and breadth of the cells.

### Growth kinetics of *C. glutamicum* strains

Bacterial growth was monitored by measuring OD at 600 nm at regular intervals until cultures reached stationary phase. *C. glutamicum* wild-type, Δ*ahfA* and Δ*ahfA* pEKEx2_cgl0396 cultures were grown in BHIS medium at 30 °C under shaking conditions. Starter cultures were first cultivated to mid-log phase (OD 600 nm ~0.6) and then diluted to an initial OD 600 nm of 0.05 in 10 ml fresh BHIS medium. Growth measurements were subsequently recorded at defined time points until stationary phase was attained. All experiments were performed at least three times independently, and the results are presented as the mean±sd.

### Lysozyme susceptibility assay

*C. glutamicum* wild-type, Δ*ahfA* mutant and Δ*ahfA* pEKEx2_cgl0396 strain were grown in BHIS medium at 30 °C with shaking to mid-log phase. Cultures were normalized to the same starting density (OD 600 nm ~0.05) in fresh BHIS medium and exposed to increasing concentrations of lysozyme (10, 50, 100 and 200 µg ml^−1^); lysozyme was added directly to the medium at the time of inoculation. Treated cultures were incubated under the same growth conditions, and bacterial growth was monitored by measuring OD 600 nm after 56 h of incubation, at which point the cultures had reached stationary phase (as established in Fig. 3c). All experiments were performed at least three times independently, and the results are presented as the mean±sd.

**Fig. 3.**
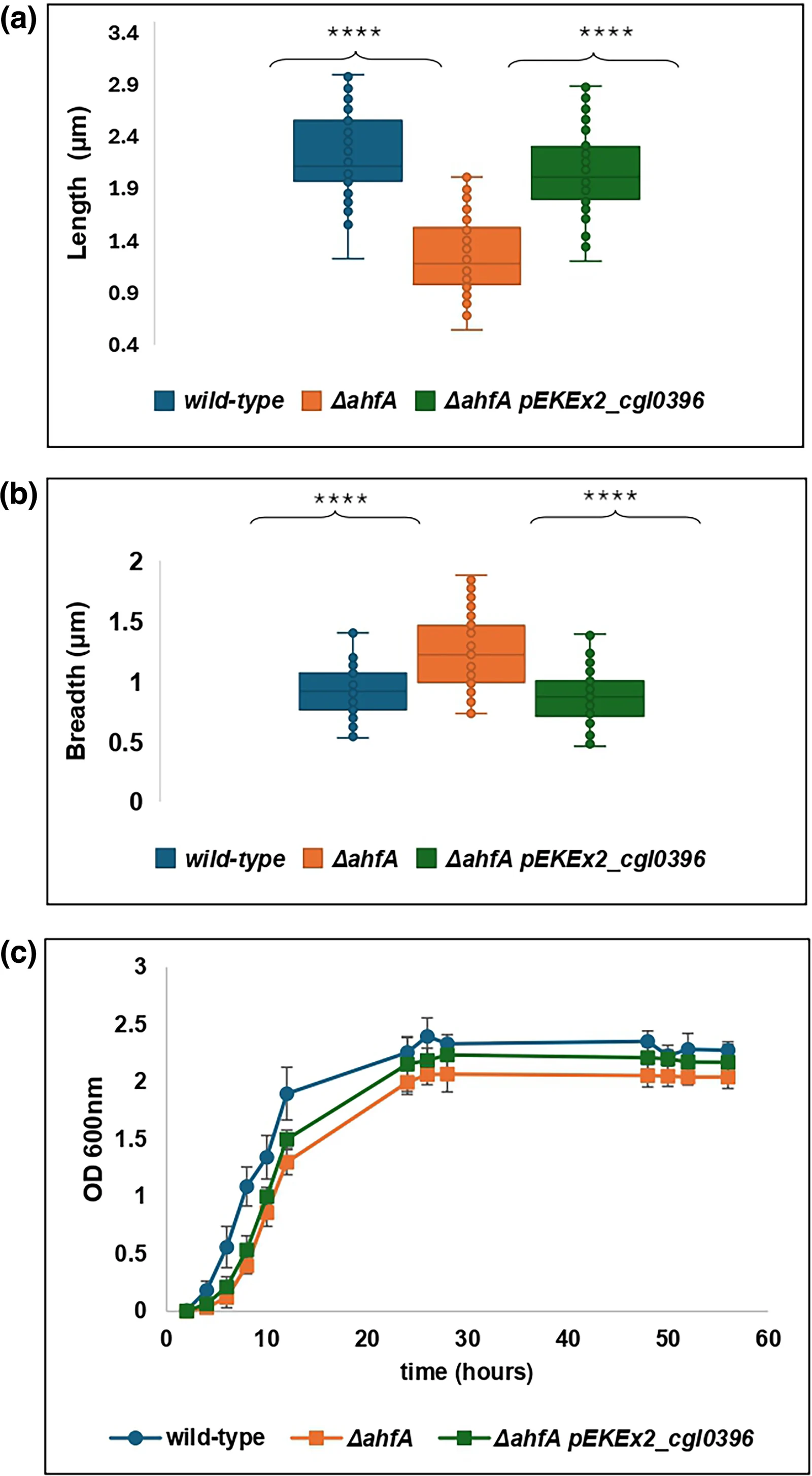
Morphological characterization and growth analysis of *C. glutamicum strains*. (**a**) Cell length measurements. (b) Cell breadth measurements of wild-type, Δ*ahfA* and Δ*ahfA* pEKEx2_cgl0396 strains measured from *n*=100 cells per strain across three independent biological replicates. Statistical significance was determined by two-tailed Welch’s t-test (****P<0.0001). (**c**) Growth curves of the strains over 56 h, showing mean±sd (*n*=3 independent biological replicates).

### Antibiotic susceptibility

Antibiotic susceptibility was evaluated using a resazurin-based [26] viability assay to determine the percentage survival of *C. glutamicum* wild-type, Δ*ahfA* and Δ*ahfA* pEKEx2_cgl0396 strain following antibiotic exposure. Rifampicin, penicillin and streptomycin stock solutions were prepared in DMSO and sterile water, respectively. Overnight cultures grown in BHIS at 30 °C were diluted to an OD 600 nm of 0.005 in fresh BHIS medium, and 20 µl was added to each well to achieve a starting OD 600 nm of 0.0005 and added to 96-well plates containing a twofold dilution series of each antibiotic. Plates were incubated statically at 30 °C for 24 h. Following incubation, 20 µl of 0.02% resazurin solution (filter sterilized in distilled water) was added to each well, and plates were incubated for an additional 1 h at 30 °C. Fluorescence was measured using a microplate reader at excitation and emission wavelengths of 560 and 590 nm, respectively. Percentage survival was calculated relative to untreated controls which were defined as 100% viability. All experiments were performed at least three times independently, and the results are presented as the mean±sd.

### Extraction and analysis of ^14^C-labelled lipids

#### Radioactive labelling

*C. glutamicum* wild-type, Δ*ahfA* and Δ*ahfA* pEKEx2_cgl0396 strain were streaked onto BHIS agar plates and incubated at 30 °C for 24 to 48 h. Single colonies were inoculated into 5 ml BHIS broth and grown overnight at 30 °C with shaking. A 1 ml aliquot of the overnight culture was used to inoculate 10 ml BHIS broth for secondary culture. Cultures were grown at 30 °C with shaking until reaching an OD 600 nm of ~0.4. At this density, cultures were labelled with 5 µCi ml^−1^ [¹⁴C]-acetate (carbon-14 labelled acetate, used as a metabolic tracer). Cultures were incubated for an additional 24 to 48 h at 30 °C before harvesting by centrifugation (4,000 r.p.m., 10 min). Pellets were washed once with PBS and dried under a gentle nitrogen stream or in a vacuum concentrator.

#### Extraction of total lipids

Total lipid extraction followed standard protocols for *Corynebacteriales* cell envelopes from Dobson *et al*. [27] and Minnikin *et al*. [28]. OM and IM lipids were isolated following the extraction protocol described by Klatt *et al*. [1]. Results are expressed from at least three independent experiments.

Densitometric analysis was conducted using ImageJ software. Following background subtraction, band intensities were quantified, with the wild-type signal normalized to 100%. Lipid abundance in the mutant strains was then determined relative to the corresponding wild-type value and expressed as a percentage of wild-type abundance. Mean values obtained from independent experiments were used for comparative analysis.

### Extraction and analysis of mycolic acid methyl esters and fatty acid methyl esters

This method follows established tetra-butyl ammonium hydroxide-based hydrolysis and methylation procedures [29, 30]. TLC was performed using Silica Gel 60 F254 plates from Merck. For the total lipid analysis, chloroform:methanol:water (60:16:2, v/v/v), 20,000 cpm (counts per minute) of each strain was used. For fatty acid methyl esters (FAMEs) and mycolic acid methyl esters (MAMEs), petroleum ether (B.P. 60–80 °C):acetone (95 : 5, v/v) 20,000 cpm was used for normal phase TLCs. For free fatty acids (FFAs) and free mycolic acids (FMAs), hexane:diethyl ether:acetic acid (70:30:1, v/v/v) 20,000 cpm of each strain was used. For 2D TLC for the apolar lipids, solvent system D as described by Dobson *et al*. [27]. To expose the [^14^C]-labelled lipids, autoradiograms were created by exposing Kodak X-Omat AR film for four to 5 days.

### Measurement of [³H]-GlcNAc incorporation into peptidoglycan

To assess cell wall incorporation of [³H]-*N*-acetylglucosamine (GlcNAc), *C. glutamicum* wild-type, Δ*ahfA* and Δ*ahfA* pEKEx2_cgl0396 strain were grown overnight in 5 ml of medium at 30 °C with shaking. They were then diluted 1:100 into 50 ml of fresh medium in a 250-ml flask and grown to the early exponential phase (OD 600 nm~0.1). [³H]-GlcNAc was added to a final activity of 5 µCi ml^−1^, and cultures were incubated for 3 to 4 generations until OD 600 nm~0.7. Cells were harvested by chilling on ice for 5 min, pelleting at 4,000 r.p.m. for 10 min and washing twice with ice-cold PBS to remove unincorporated label. Peptidoglycan sacculi were isolated by resuspending the pellet in water, adding an equal volume of 4% SDS and boiling for 30 min followed by pelleting at 4,000 r.p.m. and repeated washing with hot water until SDS was completely removed. Optional enzymatic digestion with lysozyme for 4 h at 37 °C was performed followed by additional washes. The final sacculi fraction was resuspended in 500 µl of water, and 100 µl was combined with 5 ml of scintillation cocktail (Ecoscint O National Diagnostics, which consists of a non-polar organic solvent, like alkylbenzene) for measurement of radioactivity using a liquid scintillation counter. Incorporation of [³H]-GlcNAc into the cell wall was calculated by comparing cpm in the sacculi fraction to the total cpm added to the culture with cpm in supernatants and pellets measured to account for unincorporated and cell-associated GlcNAc, respectively [31]. All experiments were performed at least three times independently, and the results are presented as the mean±sd.

## Results

### Genetic ablation of *ahfA* compromises growth and cellular architecture

Phase-contrast microscopy revealed pronounced morphological defects in *C. glutamicum* following the deletion of the *ahfA* (Fig. 4a–c). Wild-type cells exhibited the expected rod-shaped morphology with relatively consistent length and width indicative of normal cell growth and division (Fig. 4a). In contrast, the Δ*ahfA* mutant displayed a striking alteration in cell shape characterized by a significant reduction in cell length accompanied by an increase in cell breadth consistent with a shift towards a coccoid morphology (Fig. 4b), while the Δ*ahfA* pEKEx2_cgl0396 strain showed a similar phenotype as the wild-type strain (Fig. 4c).

**Fig. 4.**
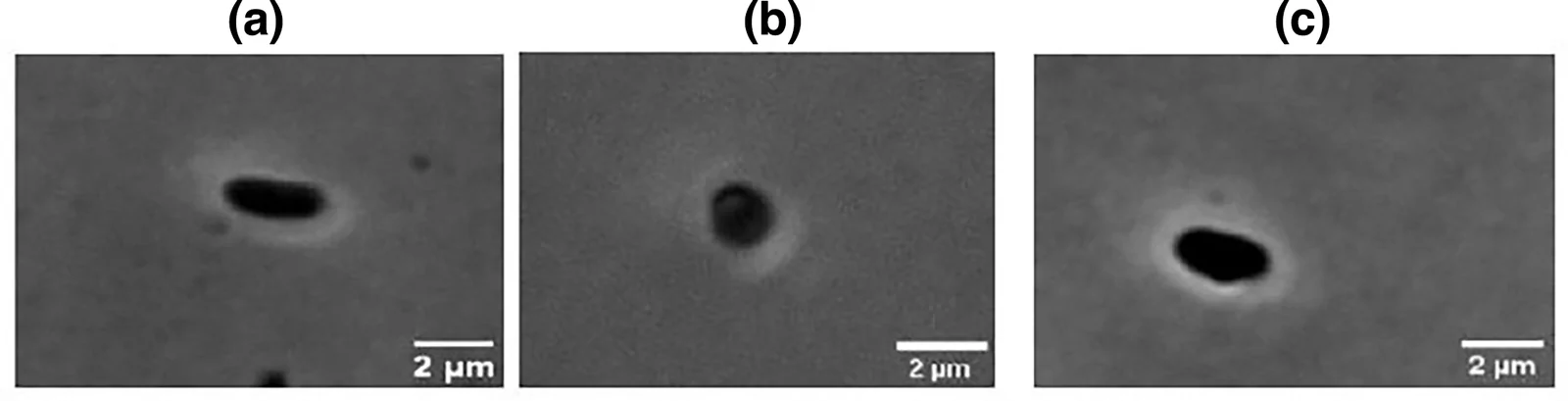
Morphological analysis of *C. glutamicum* strains using phase-contrast microscopy. Phase-contrast microscopy images showing (a) wild-type (rod shaped), (b) Δ*ahfA* (coccoid) and (c) Δ*ahfA* pEKEx2_cgl0396 (rod shaped) strain carrying *ahfA* on a plasmid. Scale bars represent 2 µm.

Quantitative morphometric analysis (Fig. 3a, b) corroborated these visual observations, showing a clear shift in the distribution of both length and breadth measurements in the mutant population compared to wild-type. The Δ*ahfA* mutant was significantly shorter than wild-type, with a median length of ~1.2 µm compared to ~2.1 µm in wild-type; a corresponding increase in breadth of the Δ*ahfA* mutant was observed, with the cells measuring ~1.2 µm compared to ~0.9 µm in the wild-type. The Δ*ahfA* pEKEx2_cgl0396 strain showed similar cell dimensions as the wild-type strain. Taken together, these changes indicate a shift towards a shorter, more spherical cell shape rather than the characteristic rod-shaped morphology of *C. glutamicum*. This is consistent with the qualitative morphological differences illustrated in Fig. 4.

Consistent with these morphological defects, growth curve analysis (Fig. 3c) revealed that the Δ*ahfA* strain exhibited a pronounced growth delay compared to wild-type, indicating that loss of *ahfA* affects cellular fitness. The Δ*ahfA* mutant, specifically, exhibited a delayed exponential growth phase and reached a slightly lower final OD 600 nm at stationary phase (~2) relative to the wild-type and Δ*ahfA* pEKEx2_cgl0396 strain (OD 600 nm of ~2.4 and 2.2, respectively). Importantly, genetic complementation of *ahfA* restored both cell morphology (Fig. 4c) and growth kinetics to near wild-type levels, with length and breadth distributions and growth profiles closely overlapping those of the wild-type strain. This complementation phenotype confirms that the observed defects in morphology and growth are directly attributable to loss of *ahfA* function rather than secondary or polar effects. These data demonstrate that *ahfA* plays a critical role in maintaining proper cell shape, size and growth in *C. glutamicum*.

### Loss of *ahfA* function compromises intrinsic antibiotic resistance and lysozyme susceptibility

Following the observation of altered morphology (Fig. 4) and the indication that cell integrity and structure were compromised in the Δ*ahfA* strain, we assessed antibiotic susceptibility. Given that *ahfA* was predicted to interact with genes involved in peptidoglycan synthesis, we focused initially on agents that specifically target peptidoglycan. Accordingly, we tested the sensitivity of the strains to lysozyme [hydrolyses β-(1,4)-glycosidic bonds of peptidoglycan] and penicillin (inhibits transpeptidation of peptidoglycan). This approach allowed us to directly evaluate whether loss of *ahfA* compromises cell wall robustness and peptidoglycan-associated resistance mechanisms.

Exposure to increasing concentrations of lysozyme, an enzyme whose access to its peptidoglycan target is restricted by the mycolic acid layer, resulted in a dose-dependent reduction in growth across all three strains (Fig. 5a). Notably, the Δ*ahfA* strain showed consistently lower growth than wild-type at every concentration tested. At the lowest concentration tested (10 µg ml^−1^), the mutant exhibits an OD 600 nm of 1.5, whereas the wild-type has an OD 600 nm of 2.25, indicating that the mutant is more vulnerable to lysozyme from the onset rather than only at higher concentrations. This gap narrowed as all three strains converged towards low growth by 200 µg ml^−1^. This phenotype was rescued by complementation, with the Δ*ahfA* pEKEx2_cgl0396 strain closely resembling wild-type across the concentration range tested.

**Fig. 5.**
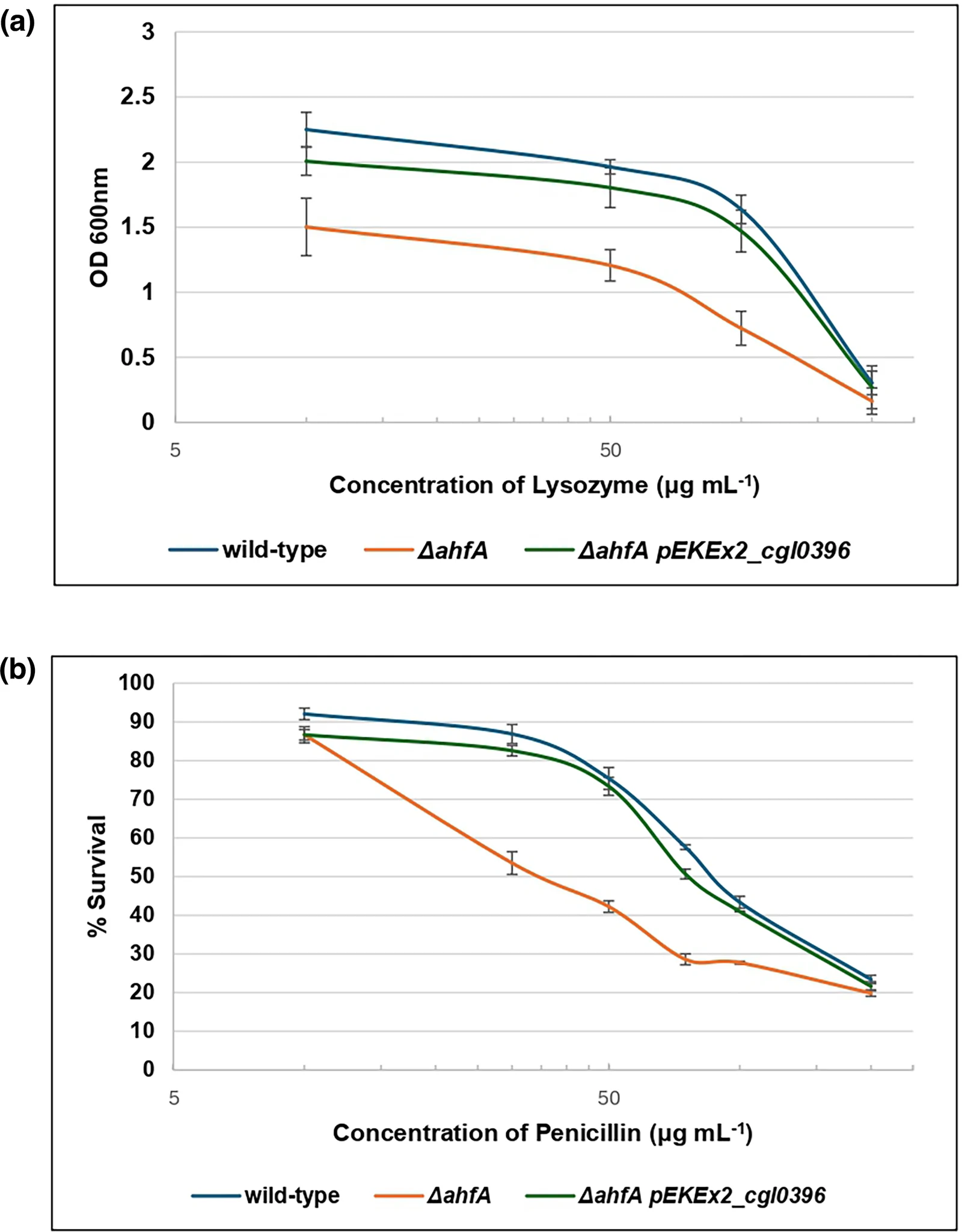
Susceptibility of *C. glutamicum* strains. (**a**) Growth of wild-type, Δ*ahfA* and the Δ*ahfA* pEKEx2_cgl0396 strain in the presence of increasing concentrations of lysozyme (10, 50, 100 and 200 µg ml^−1^), measured as OD 600 nm. (**b**) Percentage survival of wild-type, Δ*ahfA* and the Δ*ahfA* pEKEx2_cgl0396 strain following exposure to increasing concentrations of penicillin (10, 30, 50, 75, 100 and 200 µg ml^−1^), calculated relative to untreated controls. Data are presented as mean±sd (*n*=3 independent biological replicates). The x-axis is shown on a logarithmic (base 10) scale. Statistical significance was determined by two-tailed Welch’s t-test at each concentration, with Bonferroni correction for multiple comparisons; Δ*ahfA* differed significantly from wild-type at all concentrations tested for both compounds (*P*<0.005).

A related but distinct pattern was observed in penicillin (Fig. 5b) which directly acts on peptidoglycan synthesis. Unlike the lysozyme assay, all three strains showed comparable survival at the lowest concentration tested (10 µg ml^−1^), suggesting that Δ*ahfA*’s increased vulnerability is not a uniform baseline growth defect but is instead specific to the nature and concentration of the stressor. In the intermediate concentrations (30 to 75 μg ml^−1^), the Δ*ahfA* strain’s survival reduces drastically, and all three strains converged to similarly low survival at the highest concentration tested (200 µg ml^−1^). The Δ*ahfA* pEKEx2_cgl0396 strain restored almost wild-type level survival across the range.

Given that *Corynebacterium* species possess a lipid-rich cell envelope that serves as a critical permeability barrier, we evaluated the impact of *ahfA* deletion on susceptibility to antibiotics with differing physicochemical properties (lipophilic and hydrophilic). We tested two antibiotics with distinct modes of action: rifampicin (binds to RNA polymerase, inhibiting transcription) and streptomycin (inhibits protein synthesis). Rifampicin susceptibility testing revealed a pronounced increase in sensitivity in the Δ*ahfA* mutant relative to wild-type (Fig. 6a). At the lowest concentration tested (2 ng ml^−1^), wild-type cells retained ~95% survival, whereas the Δ*ahfA* strain exhibited only ~70% survival. This enhanced sensitivity was maintained across the full concentration range, with survival of the Δ*ahfA* strain remaining consistently lower than the wild-type at intermediate concentrations. At the highest concentration tested (30 ng ml^−1^), wild-type cells retained ~35% viability compared to ~20% for the Δ*ahfA* mutant. Notably, the Δ*ahfA* pEKEx2_cgl0396 strain did not fully restore wild-type level survival at higher concentrations, indicating partial rather than complete phenotypic rescue. All three strains converged to a narrower range of survival by 10 ng ml^−1^. Given that rifampicin’s mechanism does not involve the cell envelope directly, this pattern is more consistent with a permeability-mediated effect, in which a compromised mycolic acid layer in the Δ*ahfA* mutant allows greater intracellular accumulation of the lipophilic drug.

**Fig. 6.**
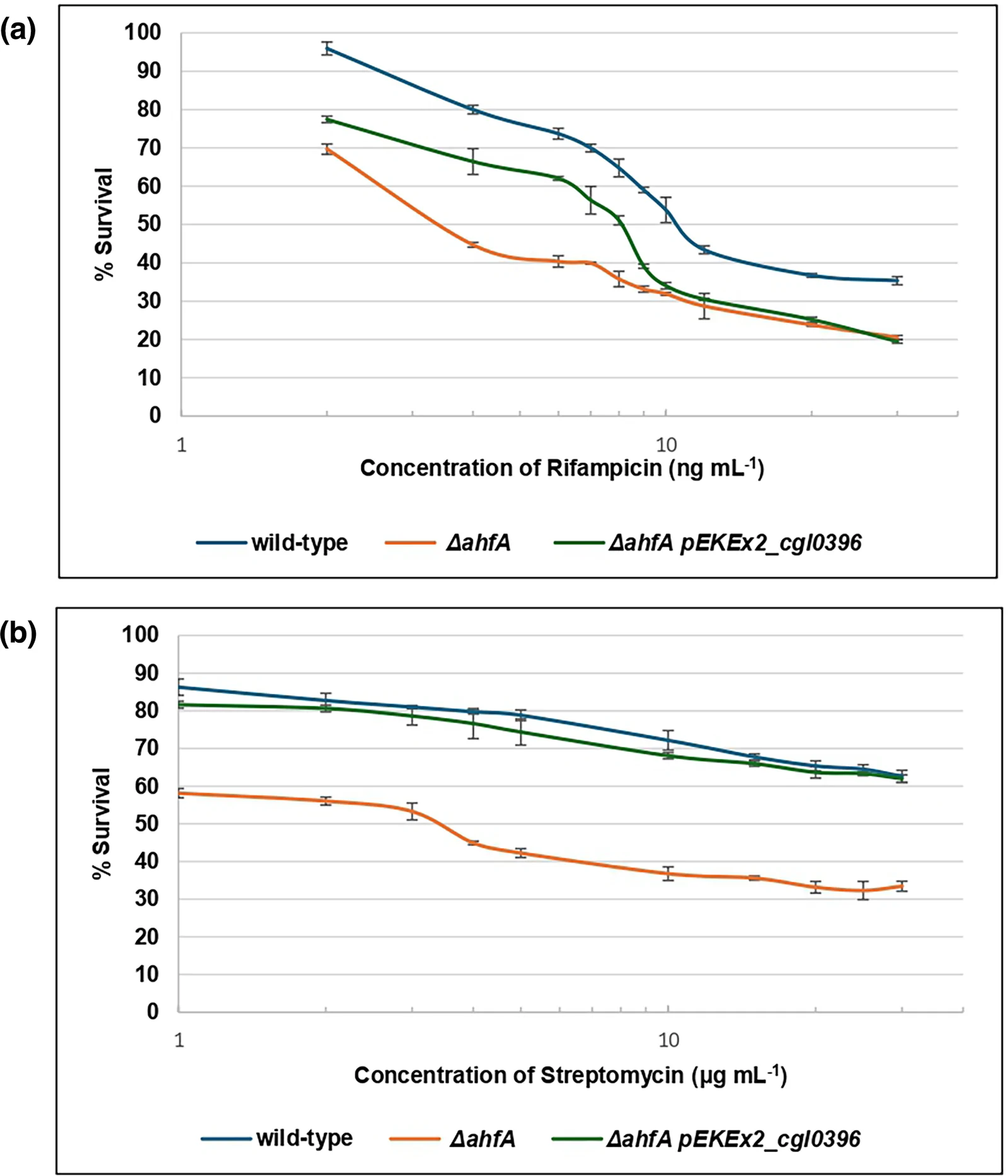
Survival of *C. glutamicum* strains under antibiotic stress. (a) Percentage survival of wild-type, Δ*ahfA* and Δ*ahfA* pEKEx2_cgl0396 strain following exposure to increasing concentrations of rifampicin (2, 4, 6, 7, 8, 9, 10, 12, 20 and 30 ng ml^−1^). (**b**) Percentage survival of wild-type, Δ*ahfA* and Δ*ahfA* pEKEx2_cgl0396 strain following exposure to increasing concentrations of streptomycin (1, 2, 3, 4, 5, 10, 15, 20, 25 and 30 µg ml^−1^). Data are presented as mean±sd (*n*=3 replicates). The x-axis is shown on a logarithmic (base 10) scale. Statistical significance was determined by two-tailed Welch’s t-test at each concentration, with Bonferroni correction for multiple comparisons; Δ*ahfA* differed significantly from wild-type at all concentrations tested for both compounds (*P*<0.005).

Streptomycin treatment produced a distinct response (Fig. 6b). The Δ*ahfA* mutant displayed a consistent reduction in viability compared to wild-type, across all concentrations tested. While wild-type cells maintained ~80% survival at 1–5 µg ml^−1^ concentration, the mutant showed a marked decrease to 45–55%. This sensitivity became more pronounced at intermediate concentrations (10–15 µg ml^−1^), where wild-type survival remained ~65–70%, compared to 35% for the Δ*ahfA* strain. At the highest concentrations (20–30 µg ml^−1^), wild-type cells retained 60–65% viability whereas the mutant plateaued at 35% indicating that the mutant remains more sensitive. The Δ*ahfA* pEKEx2_cgl0396 strain showed similar survival rates as the wild-type. Given streptomycin’s relatively poor intrinsic permeability across the mycolic acid layer, the sustained increase in susceptibility in the Δ*ahfA* mutant provides further support for a general defect in envelope permeability.

Taken together, these data show that the Δ*ahfA* mutant exhibits increased susceptibility to lysozyme and to antibiotics with distinct targets and also different physicochemical properties, a pattern more consistent with a generalized defect in the cell envelope permeability.

### Δ*ahfA* mutant exhibits pleiotropic defects in lipid biosynthesis and composition

To elucidate the molecular basis underlying the altered morphology and antibiotic susceptibility of the Δ*ahfA* mutant, we analysed the lipid composition of wild-type, Δ*ahfA* and the Δ*ahfA* pEKEx2_cgl0396 strains. TLC revealed pronounced differences in lipid profiles between the strains. Specifically, the Δ*ahfA* mutant displayed a substantial reduction in both trehalose monocorynomycolate (TMCM) and trehalose dicorynomycolate (TDCM) relative to the wild-type (Fig. 7a). In addition to the loss of TMCM and TDCM, a novel lipid species was detected in the mutant at an intermediate Rf position that was absent from the wild-type strain. Complementation partially restored the lipid profiles with TMCM and TDCM levels to those observed in the wild-type.

**Fig. 7.**
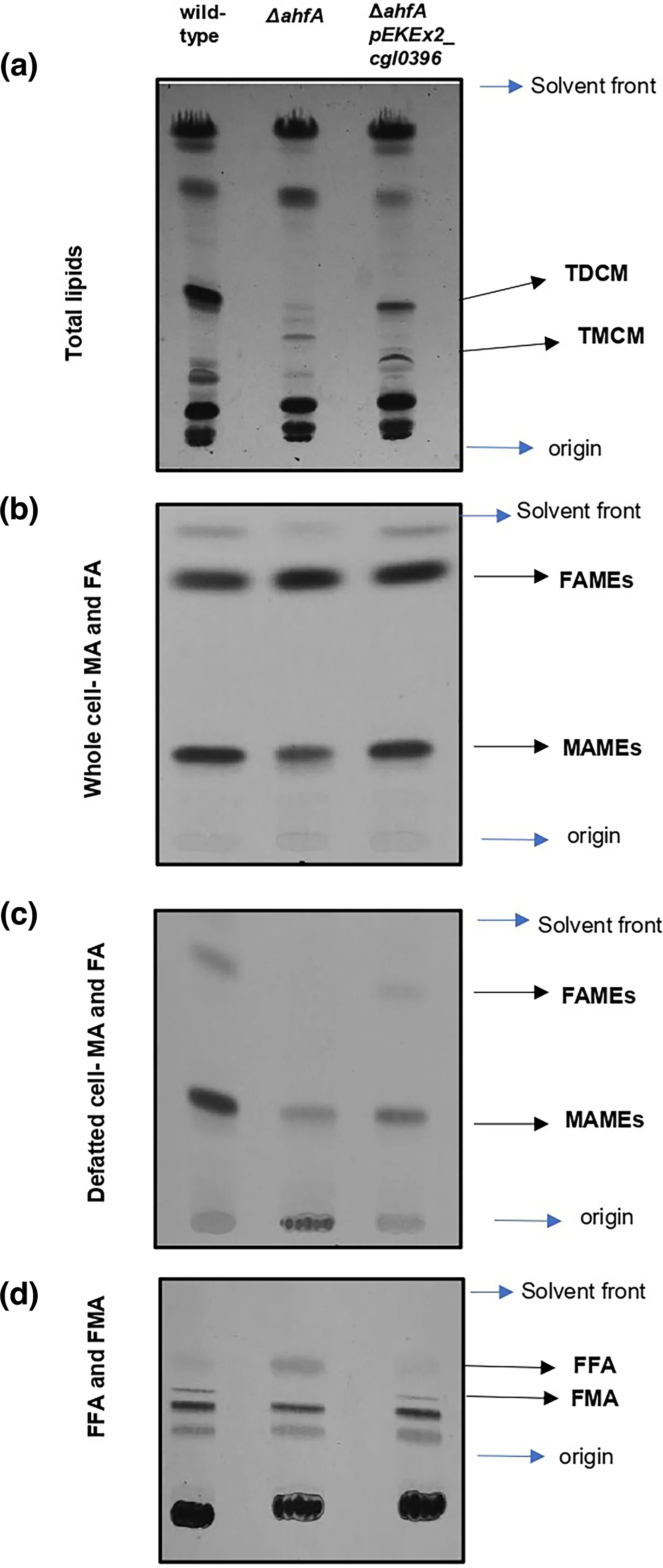
TLC of ¹⁴C-radiolabelled lipid fractions. (**a**) Total lipid extract showing the complete profile of radiolabelled lipid species. (b) Whole-cell MAMEs and FAMEs. (c) Defatted cell MAMEs and FAMEs. (d) FFAs and FMAs.

Given that AhfA has been predicted to function in mycolic acid biosynthesis, we examined the mycolic acid content in both whole cells (bound mycolic acids as MAMEs, TDCM and TMCM) and defatted cells (only bound mycolic acids as MAMEs). TLC of the whole cells (Fig. 7b) revealed that MAMEs across wild-type, Δ*ahfA* and the Δ*ahfA* pEKEx2_cgl0396 strains are present, with visually lower levels in the mutant. All three strains exhibited clearly resolved FAMEs. The Δ*ahfA* mutant showed increased FAME levels, whereas the Δ*ahfA* pEKEx2_cgl0396 strain displayed levels broadly comparable to those of the wild-type. These findings indicate that the fatty acid biosynthesis remains intact in the Δ*ahfA* mutant.

Analysis of defatted cell preparations (Fig. 7c) further revealed differences in the covalently bound lipid fraction, with the bound mycolic acid levels significantly reduced in the Δ*ahfA* mutant. Complementation of the mutant partially restored the wild-type phenotype. The FAME band was detected at the expected position in the wild-type defatted fraction. In contrast, the band was completely absent in the Δ*ahfA* mutant and was partially restored in the Δ*ahfA* pEKEx2_cgl0396 strain. Taken together, the reduction in cell wall bound mycolic acids, TMCM and TDCM in the Δ*ahfA* mutant suggests that AhfA specifically disrupts mycolic acid biosynthesis itself.

In corynebacteria, FFAs and free FMAs function as key intermediates for the biosynthesis of complex cell envelope lipids. FMAs are typically transferred to trehalose acceptors to generate TMCM and TDCM, which are essential components of the OM, while FFAs serve as precursors for mycolic acids as well as other membrane-associated lipids, including phospholipids and acylated glycolipids. Analysis of lipid fractions (Fig. 7d) from the Δ*ahfA* mutant revealed a pronounced accumulation of FFAs accompanied by a reduction in FMAs compared with the wild-type strain; the Δ*ahfA* pEKEx2_cgl0396 strain could rescue this phenotype and was comparable to the wild-type. Taken together, these findings suggest that rather than accumulating as a free, non-trehalose linked pool, mature mycolic acids produced in the mutant do not persist as a detectable free intermediate. Instead, they may be rapidly diverted towards an alternative fate, potentially contributing to the unidentified lipid species detected in the total lipid profile (Fig. 7a). The substantial increase in FFA levels further indicates an accumulation of fatty acid precursors following disruption of mycolic acid biosynthesis, possibly reflecting the build-up of FAS-I-derived intermediates that are not efficiently channelled into the mycolic acid biosynthetic pathway.

To distinguish whether *ahfA* affects trehalose mycolate synthesis or their incorporation into the cell envelope, lipids were extracted from isolated IM and OM fractions. TLC (Fig. 8a, b) revealed that TMCM and TDCM were absent from both membrane fractions in the Δ*ahfA* mutant, whereas these lipids were readily detected in OM fractions of the wild-type and Δ*ahfA* pEKEx2_cgl0396 strains. Notably, if AhfA were solely involved in lipid transport, an accumulation of TMCM and TDCM in the IM with a corresponding depletion in the OM would be expected. Instead, the complete absence of these lipids in both fractions indicates that the defect occurs upstream of transport at the level of early stages of mycolic acid biosynthesis. These findings rule out a primary role for AhfA in trehalose mycolate translocation.

**Fig. 8.**
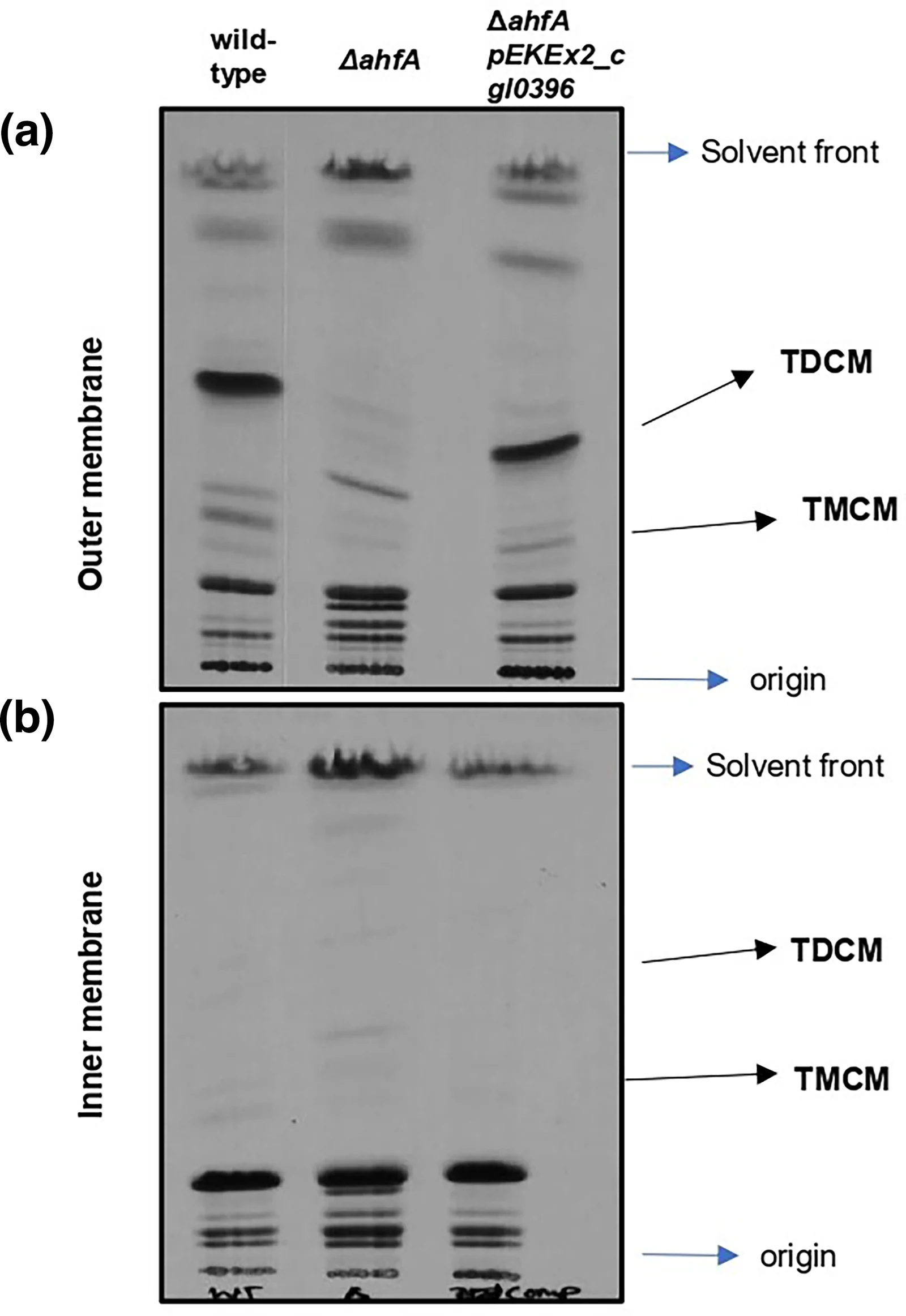
TLC analysis of ¹⁴C-radiolabelled lipid subfractions isolated from distinct cellular compartments. (**a**) OM lipids. (b) IM lipids.

### AhfA function in lipid metabolism is conserved in mycobacteria

Given the pronounced reduction in TMCM and TDCM observed in the *Corynebacterium* Δ*ahfA* mutant, we next examined whether deletion of *ahfA* orthologues in mycobacteria produced similar effects on lipid composition. 2D TLC of the apolar lipid fractions (Fig. 9a, b) of the corresponding mycobacterial mutants revealed detectable differences compared to their respective wild-type strains.

**Fig. 9.**
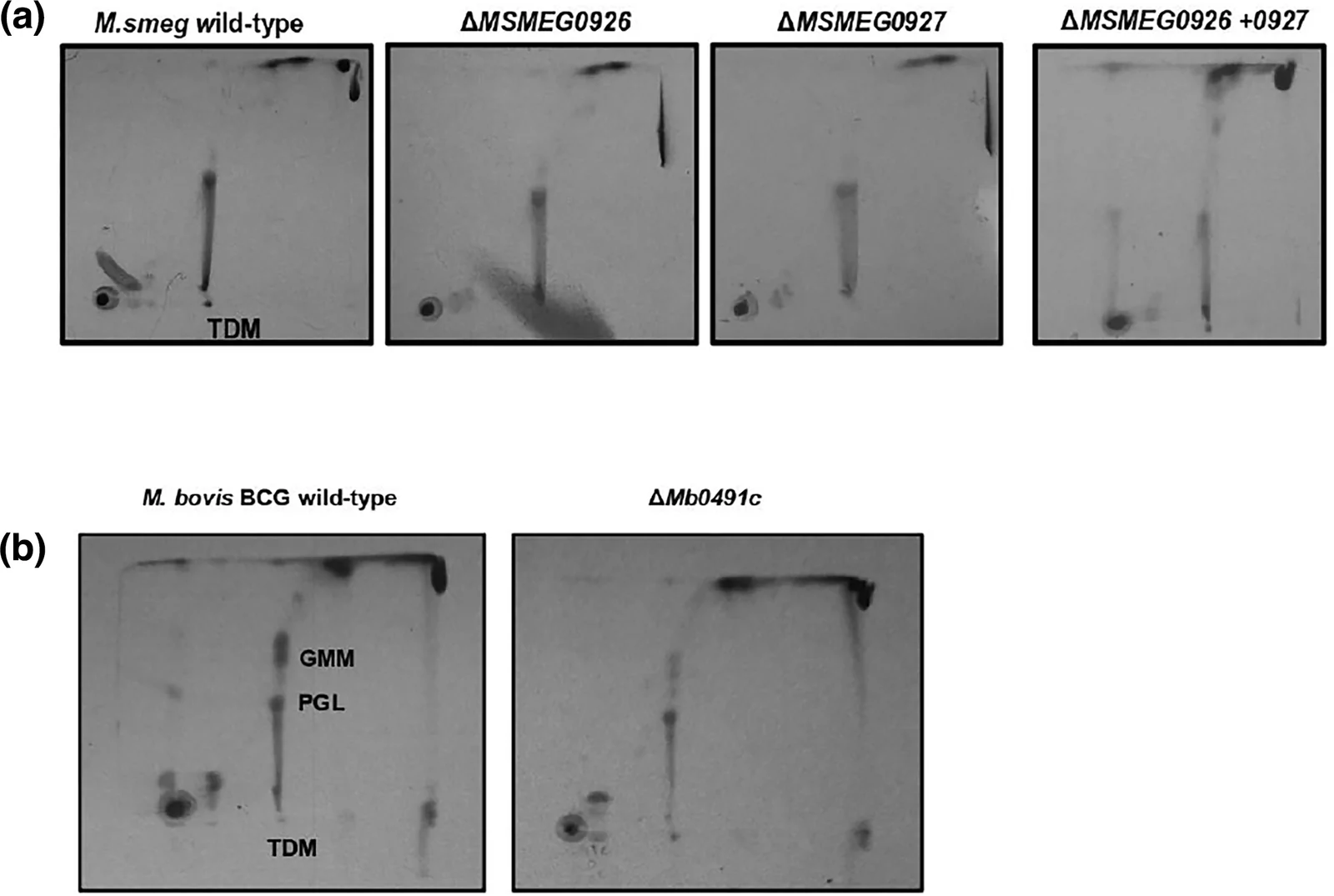
: 2D TLC analysis of lipid extracts from *Mycobacterium* strains. (**a**) Lipid extracts from *M. smegmatis* and Δ*MSMEG0926*, Δ*MSMEG0927*, Δ*MSMEG0926*+Δ*MSMEG0927* mutants. (b) Lipid extracts from *M. bovis* BCG wild-type and Δ*Mb0491c* mutant.

Analysis of the apolar lipid fractions from Δ*MSMEG0927* and Δ*MSMEG0926*+Δ*MSMEG0927* strains (Fig. 9a) showed a visible reduction in TDM levels compared with the wild-type and Δ*MSMEG0926* strains. Densitometric analysis confirmed a decrease in the relative abundance of TDM by 11.3% in the Δ*MSMEG0927* mutant and 9.8% in the Δ*MSMEG0926*+Δ*MSMEG0927* mutant. These findings indicate that the deletion of *MSMEG_0927* results in a phenotype similar to *Corynebacterium*, particularly with respect to the TDM abundance.

Analysis of the apolar lipid fractions from *M. bovis* BCG resolved TDM and glucose monomycolate (GMM) species (Fig. 9b). Compared with the wild-type strain, the Δ*Mb0491c* mutant exhibited reduced levels of both TDM and GMM. Densitometric analysis confirmed decreases of 14.3 and 20.2% in the relative abundance of TDM and GMM, respectively, in the Δ*Mb0491c* mutant.

Taken together, deletion of the *ahfA* orthologues, *MSMEG0927* and *Mb0491c*, resulted in detectable alterations in the apolar lipid profile, similar to the *Corynebacterium* mutant. The decrease in TDM was observed across both mycobacterial species, with an additional reduction in GMM in the Δ*Mb0491c* mutant. GMM is a mycolic acid-containing glycolipid. These findings suggest that AhfA orthologues contribute to the normal production or maintenance of mycolic acid-containing lipids in mycobacteria.

### Δ*ahfA* mutation impairs cell envelope permeability

Given the broad phenotypic defects observed in the Δ*ahfA* mutant including altered growth, increased antibiotic susceptibility and disrupted cell envelope lipid composition in *C. glutamicum,* we investigated whether these changes were accompanied by defects in cell envelope permeability. In addition, because AhfA is predicted to interact with genes involved in peptidoglycan synthesis, we assessed uptake of radiolabelled [^3^H]-*N*-acetylglucosamine (GlcNAc). GlcNAc is a key precursor for peptidoglycan biosynthesis, and its uptake depends on effective permeability and transport across the envelope [32, 33]. Measuring GlcNAc uptake will allow us to determine whether these envelope defects also impact the ability of the cell to import essential building blocks, providing a functional readout of overall envelope integrity. Strikingly, the Δ*ahfA* mutant exhibited an ~48% reduction in ^3^H-GlcNAc uptake (Fig. 10) as compared to the wild-type strain, indicating a pronounced impairment in cell envelope permeability. Complementation of the mutant fully restored GlcNAc uptake to wild-type levels, confirming that this defect is directly attributable to the loss of AhfA function. Reduced uptake of GlcNAc, a key precursor for cell wall biosynthesis, implies that envelope permeability defects may limit the access of essential metabolites required for cell wall assembly and maintenance.

**Fig. 10.**
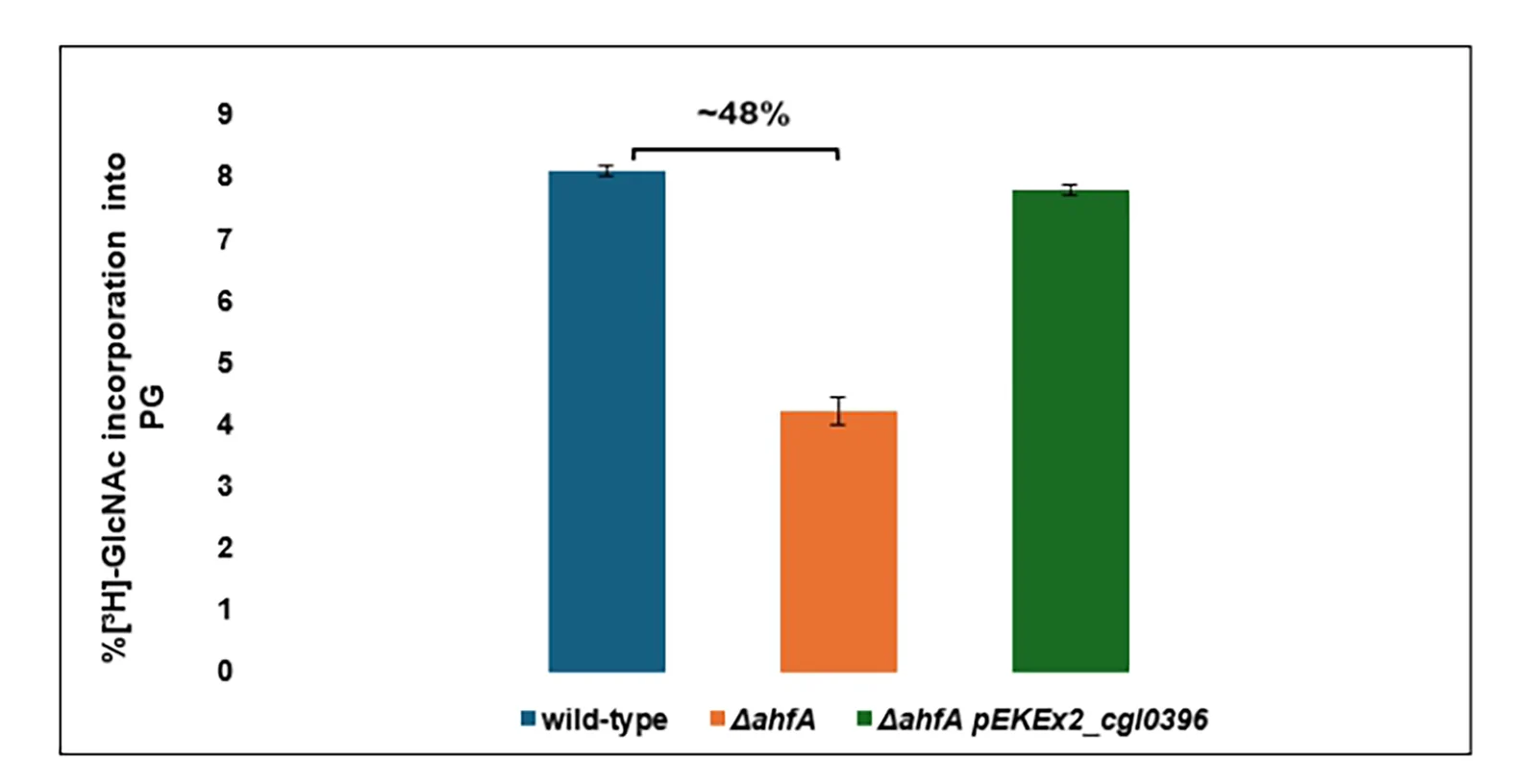
Measurement of [³H]-GlcNAc incorporation into peptidoglycan in *C. glutamicum* strains.

## Discussion

This study identifies AhfA as an important contributor to cell envelope organization in *C. glutamicum*. Deletion of *ahfA* produced a broad phenotype affecting cell morphology, growth, antibiotic susceptibility, permeability and lipid composition, indicating that AhfA plays a central role in maintaining mycolic acid homeostasis and cell envelope integrity. Comparative analysis of AhfA orthologue deletion mutants in *M. smegmatis* and *M. bovis* BCG demonstrated that aspects of the *C. glutamicum* phenotype are conserved in mycobacteria, raising the possibility that AhfA function could be conserved across the order *Corynebacteriales*.

Loss of *ahfA* caused pronounced morphological changes with mutant cells appearing shorter and wider than the wild-type. Such alterations are commonly associated with defects in envelope biogenesis. The accompanying growth delay further supports the notion that AhfA is required for optimal cellular fitness. Importantly, complementation restored both morphology and growth kinetics, demonstrating that these defects are specifically due to the absence of AhfA and not secondary mutations. These findings highlight the close relationship between envelope architecture and fundamental cellular processes in *C. glutamicum*.

The increased susceptibility of the Δ*ahfA* mutant to both rifampicin and streptomycin, together with its enhanced sensitivity to lysozyme, provides evidence that loss of AhfA compromises the integrity and barrier function of the *Corynebacterium* cell envelope. The effect was observed for compounds with distinct physicochemical properties and different intracellular targets which supports alteration in the overall envelope permeability. The pronounced sensitivity to rifampicin is consistent with increased penetration of the lipophilic antibiotic as a consequence of alterations in the mycolic acid layer. Although streptomycin is less intrinsically permeable across the lipid-rich envelope, the sustained increase in susceptibility of the Δ*ahfA* mutant suggests that the envelope defect extends beyond the disruption of the mycolic acid layer. The Δ*ahfA* mutant displayed increased susceptibility to lysozyme and penicillin, suggesting that the changes in the outer lipid layers compromise the protective function of the cell envelope and render the Peptidoglycan layer more accessible to damage. These findings support a role of AhfA in maintaining the structural and functional properties of the mycolic acid layer.

Lipid profiling demonstrated that the Δ*ahfA* mutant exhibits a near complete loss of cell wall bound MAMEs, together with a marked depletion of TMCM and TDCM, indicating a profound disruption of mycolic acid biosynthesis and incorporation into the cell envelope. This phenotype resembles that observed following disruption of the essential mycolic acid biosynthetic enzyme, Pks13, where both cell wall-bound MAMEs and trehalose-linked mycolates are severely depleted. One possibility is that AhfA may functionally interact with Pks13 or another component of the mycolic acid biosynthetic pathway which leads to disruption in overall production of mycolic acid and limits the formation of outer envelope glycolipids. Analysis of the defatted cell fractions further supports this interpretation, demonstrating a substantial reduction in covalently cell wall bound mycolic acids in the Δ*ahfA* mutant. The partial recovery of the defatted cell MAMEs following complementation was consistent with the partial restoration of TMCM and TDCM.

The marked accumulation of FFA in the Δ*ahfA* mutant further suggests that fatty acid precursors accumulate upstream of mycolic acid maturation when downstream biosynthetic flux is impaired. This may reflect a feedback effect whereby disruption of mycolic acid biosynthesis leads to the accumulation of precursor fatty acids that cannot be efficiently channelled into the downstream pathway. The absence of detectable accumulation of FMA provides an additional insight; if AhfA was required for the esterification of mature mycolic acids to trehalose, an accumulation of FMA would be expected. Instead, the lack of detectable FMA suggests that mycolic acids generated in the mutant are rapidly diverted towards an alternative metabolic fate, probably the unidentified lipid species in the total lipid profile. The absence of detectable TMCM and TDCM accumulation in the IM fraction of the Δ*ahfA* mutant indicates that AhfA is unlikely to be directly involved in the transport of these trehalose-linked mycolates. Furthermore, this finding supports the conclusion that the depletion of these glycolipid species reflects a true reduction in their production rather than their redistribution.

The imbalance in lipid intermediates observed in the mutant indicates that normal metabolic flux is interrupted, leading to accumulation of precursors that cannot be efficiently incorporated into the cell envelope. Functionally, this disruption manifests as altered permeability demonstrated by reduced uptake of GlcNAc. The 48% reduction in incorporation represents a substantial decrease and may indicate an underlying defect in PG biosynthesis. One possible explanation is the genomic proximity of the *ahfA* locus to genes involved in PG biosynthesis. However, it remains unclear whether AhfA directly affects *murB* expression through transcriptional regulation or whether the observed reduction in PG synthesis results from an indirect or more complex regulatory mechanism. Further investigation including direct assessment of PG cross-linking and quantitative analysis of *murB* expression will be required to determine the molecular basis of this phenotype.

The reduction in TDM (and GMM in *M. bovis* BCG) observed in the mycobacterial mutants is consistent with the lipid phenotype of the *C. glutamicum* Δ*ahfA* mutant. In *M. smegmatis*, the phenotype associated with loss of *MSMEG_0927* may reflect functional redundancy with other proteins like *MSMEG_0926*. In contrast, the reduction in mycolic acid-containing lipids observed in the Δ*Mb0491c* mutant suggests that comparable functional redundancy may be limited in *M. bovis* BCG. Collectively, the alterations in TDM and GMM in mycobacteria indicate that the AhfA orthologues contribute to the maintenance of mycolic acid homeostasis. These findings further support a conserved role for the AhfA protein family in mycolic acid metabolism and consequently in maintaining the integrity of the mycobacterial cell envelope.

Based on the data, the most plausible working model is that AhfA functions at an early stage of mycolic acid biosynthesis, potentially in association with Pks13, upstream of the trehalose esterification step. Loss of AhfA appears to disrupt mycolic acid maturation or production before trehalose esterification, resulting in the depletion of TMCM and TDCM, therefore reducing the mycolylation of the cell envelope. However, whether AhfA functions as a catalytic protein, structural scaffold or regulatory factor remains unresolved. Defining its precise biochemical role will require further characterization of the purified protein, including structural analysis and *in vitro* activity assays. Furthermore, the genomic association of *ahfA* with *murB* raises the possibility of an additional connection to PG biosynthesis, and future studies will be necessary to distinguish direct effects of AhfA loss from secondary consequences.

The compromised envelope of the *C. glutamicum* Δ*ahfA* mutant, characterized by increased susceptibility to rifampicin, streptomycin, penicillin and lysozyme, demonstrates that loss of AhfA results in broad vulnerability to antimicrobial and cell envelope-associated stresses. This phenotype highlights the potential therapeutic relevance of the AhfA pathway. If a functionally equivalent AhfA protein is present in *M. tuberculosis*, inhibition of this pathway could potentially disrupt mycolic acid-containing outer envelope lipids, TMM and TDM, which is essential for survival in *M. tuberculosis*. Overall, our findings identify AhfA as a previously uncharacterized gene which contributes to mycolic acid homeostasis and cell envelope integrity within the order *Corynebacteriales* and provide a framework for further investigation of its molecular function and potential as a therapeutic target.

## Abbreviations

AES: allelic exchange substrate
BHIS: Brain Heart Infusion Soribtol
cpm: counts per minute
FAMEs: fatty acid methyl esters
FAS-I: fatty acid synthase I
FFA: free fatty acid
FMA: free mycolic acid
GMM: glucose monomycolate
IM: inner membrane
MAMEs: mycolic acid methyl esters
OM: outer membrane
TDCM: trehalose dicorynomycolate
TMCM: trehalose monocorynomycolate
